# The Function of Paraventricular Thalamic Circuitry in Adaptive Control of Feeding Behavior

**DOI:** 10.3389/fnbeh.2021.671096

**Published:** 2021-04-27

**Authors:** Gorica D. Petrovich

**Affiliations:** Department of Psychology and Neuroscience, Boston College, Chestnut Hill, MA, United States

**Keywords:** arousal, energy homeostasis, feeding, interoception, stress, taste, viscerosensory

## Abstract

The paraventricular nucleus of the thalamus (PVT) is a complex area that is uniquely embedded across the core feeding, reward, arousal, and stress circuits. The PVT role in the control of feeding behavior is discussed here within a framework of adaptive behavioral guidance based on the body’s energy state and competing drives. The survival of an organism depends on bodily energy resources and promotion of feeding over other behaviors is adaptive except when in danger or sated. The PVT is structurally set up to respond to homeostatic and hedonic needs to feed, and to integrate those signals with physiological and environmental stress, as well as anticipatory needs and other cognitive inputs. It can regulate both food foraging (seeking) and consumption and may balance their expression. The PVT is proposed to accomplish these functions through a network of connections with the brainstem, hypothalamic, striatal, and cortical areas. The connectivity of the PVT further indicates that it could broadcast the information about energy use/gain and behavioral choice to impact cognitive processes—learning, memory, and decision-making—through connections with the medial and lateral prefrontal cortical areas, the hippocampal formation, and the amygdala. The PVT is structurally complex and recent evidence for specific PVT pathways in different aspects of feeding behavior will be discussed.

## Introduction

The paraventricular nucleus of the thalamus (PVT) is a complex, multimodal area that is uniquely embedded across the core feeding, reward, arousal, and stress circuits (Hsu et al., 2014; Colavito et al., 2015; Millan et al., 2017). Its structural position and connectivity enables it to direct feeding behavior in response to physiological, cognitive, hedonic, and environmental signals and perturbations (Millan et al., 2017; Petrovich, 2018a). The PVT can regulate both food foraging (seeking) and consumption and may balance their expression. These regulations occur under the prominent influence of bodily internal (interoceptive) signals. Here, it is conceptualized that the PVT core function is to ensure animal’s survival—promoting behaviors that avoid starvation and danger, and balancing foraging against threats and other competing behaviors. Together with guiding behavioral expression, the PVT is set up to broadcast the information about the behavioral choice and energy gain/loss, and accordingly impact cognitive processes—learning, memory, and decision-making. It could accomplish this within a complex and widespread network of connections with brainstem, hypothalamic, striatal, and cortical areas, including the ventral subiculum and CA1 within the hippocampal formation (Thompson and Swanson, 2003; Cenquizca and Swanson, 2006; Kirouac, 2015; Vertes et al., 2015). Determining the function of specific PVT pathways has been the focus of recent investigations and will be discussed here in the context of feeding behavior.

## PVT in Adaptive Behavioral Control: Prioritizing Feeding Over Other Behaviors, Except When in Danger

Hunger and stress are primary survival threats and the PVT guides behavioral choice in response to each and when they compete. Because energy is essential for survival, feeding is prioritized over other behaviors, except when an animal is in danger or when there is a sufficient surplus of energy. Adaptive control of feeding behavior, therefore, involves balancing hunger versus other competing drives and resolving their priorities. Indeed, the PVT is critical for appropriate behavioral selection in conflict settings when an animal needs to choose between competing behaviors: food seeking versus threat avoidance (Choi and McNally, 2017) or when an ambiguous cue signals both (Choi et al., 2019).

**FIGURE 1:**
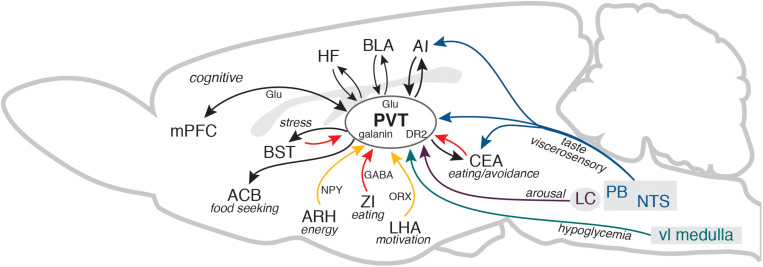
The diagram illustrates several key PVT connections discussed in the manuscript in the context of adaptive control of feeding behavior. For clarity, some connections between the areas, other brains areas that participate in the circuitry, and different PVT neurons that contain specific peptides or receptors are not shown. Cortical and thalamic glutamatergic pathways (Glu) are shown in black and GABA pathways from the ZI, CEA, and BST are shown in red. AI, agranular insular cortex; ACB, nucleus accumbens; ARH, arcuate nucleus of the hypothalamus; BLA, basolateral area of the amygdala; BST, bed nuclei of the stria terminalis; CEA, central nucleus of the amygdala; DR2, dopamine receptor 2; HF, hippocampal formation (ventral CA1 and subiculum); LC, locus coeruleus; LHA, lateral hypothalamic area; mPFC, medial prefrontal cortex; NPY, neuropeptide Y; NTS, nucleus of the solitary tract; ORX, orexin/hypocretin; PB, parabrachial nucleus; PVT, paraventricular nucleus of the thalamus; vl medulla, ventrolateral medulla; ZI, zona incerta. The sagittal outline of the rat brain was adapted from Swanson (2018).

A unique feature of the PVT is that it is well positioned to integrate the information about animal’s energy state and external prospects for gaining or depleting energy. In addition to energy, hunger and satiety signals, including dense innervation by orexigenic and anorexigenic neuropeptides, the PVT contains neurons that are sensitive to glucose and receives information about the bodily glucose state (Labouèbe et al., 2016; Beas et al., 2020). The PVT can also receive information about pending energy expenditures, such as approaching danger, stress, or changes in wakefulness/arousal states. It receives stress and arousal information from the brainstem and hypothalamus and it is interconnected with the medial (prelimbic and infralimbic) and lateral (insular) prefrontal cortical areas, the hippocampal formation, and the amygdala (Kirouac, 2015). Thus, the PVT is well positioned to detect when energy totals change or are expected to change, and guide behavioral outcome accordingly, along with recruiting arousal, while simultaneously broadcasting that information via cognitive (cortical) systems (Figure 1).

## The PVT Circuitries for Food Seeking and Consumption

Depletion of energy and other homeostatic signals drive food seeking behaviors and consumption. Cues associated with food and feeding location, or memory of hedonic or aversive post-ingestive effects, can also drive these behaviors in the absence of hunger (Petrovich, 2018a). The connections of the PVT indicate that it could receive these multifaceted signals and in turn control both food seeking and consumption and may be balancing their expression. Distinct and potentially competing PVT circuitries appear to regulate these two behaviors. Food seeking involves planning, navigation, risk assessment, and learning and memory, and PVT’s connections with the prefrontal cortex, hippocampal formation, and the amygdala would enable these computations (Kirouac, 2015). Thus the PVT is well positioned to bias food seeking over other behaviors, and to re-direct it to consumption when food is found, or avoidance when faced with danger.

### Food Consumption: Homeostatic, Hedonic, and (No)Feeding Under Stress

Early evidence that the PVT is critical in the control of food consumption came from lesion and inactivation studies (Bhatnagar and Dallman, 1999; Stratford and Wirtshafter, 2013). These studies showed that shutting down PVT drives food consumption. The rodent PVT does not contain GABA neurons (Arcelli et al., 1997), however, GABAergic inputs from the zona incerta were shown to powerfully drive consumption, particularly of high-energy (high fat) food (Zhang and van den Pol, 2017). Fasting and ghrelin increased activity of that pathway, and repeated photostimulations caused weight gain, indicating a role in homeostatic regulation. Other substantial GABA inputs to the PVT include forebrain and brainstem areas that could provide multiple ways to stimulate feeding (Kelley et al., 2005; Petrovich, 2018a,b; Otis et al., 2019). Notably, hypothalamic AgRP neurons, which also express NPY and GABA, mediate rapid food consumption via GABA or NPY release (Krashes et al., 2013). In addition, the central nucleus of the amygdala (CEA) is well positioned to mediate cognitive, stress, and hedonic effects on feeding potentially together with the PVT (Petrovich, 2018a).

In addition to direct inputs, the CEA and PVT are interconnected via multiple relays (Kirouac, 2015; Vertes et al., 2015; Petrovich, 2018a). The CEA and PVT connections have been highlighted in regard to stress (Hsu et al., 2014) and the PVT-CEA pathway is critical for fear memory acquisition and retrieval (Do-Monte et al., 2015; Penzo et al., 2015). In the context of feeding behavior, the CEA is necessary for inhibition of feeding under fear (Petrovich et al., 2009; Reppucci and Petrovich, 2018), and its neurons that express protein kinase C-delta drive anorexic effects in response to diverse aversive events (Cai et al., 2014). Intriguingly, the CEA also has stimulatory effects and drives hedonic eating but via distinct neurons that express serotonin receptor 2a (Douglass et al., 2017) and prepronociceptin (Hardaway et al., 2019). Whether these different groups of neurons are interconnected with the PVT and how these connections are organized is an important question for future research. Indeed, the CEA-PVT system may be an important site of dysregulation that could cause unnecessary food avoidance, or excessive hedonic eating.

The CEA and PVT are well positioned to impact palatability and hedonic eating, as both are prominently involved in processing taste and visceral sensory information (Thompson and Swanson, 2003; Kirouac, 2015). Changes in taste perception intensity could underlie maladaptive overeating or under-eating, such as when palatable tastes are intensified to become irresistible or when they are diminished, due to anhedonia or stress, and nothing tastes good. Whether such malfunctions would occur with overactive or underactive CEA–PVT circuitry remains to be investigated. Another important question is whether that system functions differently in males and females, as there are sex differences in hedonic and eating disorders (Culbert et al., 2021; Quigley et al., 2021). Nevertheless, as discussed next, there is support for the role of the CEA and the thalamus (possibly including PVT) in a neural circuitry underlying individual differences in taste perception.

A recent study by Veldhuizen, Small and colleagues (Veldhuizen et al., 2020) showed that the CEA responses in humans were correlated with intensity ratings across multiple tastants and that these individual differences were also reflected in activation patterns in the thalamus. Their psychophysiological and dynamic causal modeling analyses suggested that inhibitory inputs from the amygdala to the thalamus act as a central gain mechanism that influences taste intensity perception. An intriguing possibility is that the PVT was among thalamic nuclei identified in that study. The activity patterns were concentrated in the mediodorsal and ventral posterior medial thalamic areas that span the PVT location. Thus, it is possible that the PVT contributed to the observed thalamic activity in that study, but was not specifically detected due to its size and limits of fMRI resolution. The PVT is interconnected with the CEA and insular cortex, and could be a missing link in the observed functional circuitry from the CEA to the insular cortex and to the ventral posterior medial thalamus, where the CEA impacted activity without direct connections (Veldhuizen et al., 2020).

#### PVT Circuitries Shutting Down Feeding When Sated or Under Stress

When sated or when faced with danger, it is adaptive to stop feeding and prioritize other behaviors. In accordance, activation of anorexigenic GLP-1 (glucagon-like peptide-1) receptors in the PVT decreased food intake and seeking behaviors (Ong et al., 2017). Given that inhibitory inputs to the PVT drive consumption, excitatory inputs or disinhibition of the PVT projecting neurons should shut down feeding. The ventromedial nucleus of the hypothalamus (VMH) is considered to inform the PVT during states of energy surplus, within its broader output to normalize homeostasis, and activation of glutamatergic inputs to the PVT from the VMH SF1 neurons (expressing steroidogenic factor 1) were shown to suppress food intake (Zhang et al., 2020). Interestingly, that manipulation did not impact metabolism, indicating that the VMH-PVT pathway may exclusively regulate the behavioral component of energy balance.

Danger shuts down feeding and stress-related signals should impact the PVT in the opposite direction from signals that drive feeding. Consistent with this notion, under stress, the locus coeruleus dopaminergic pathway has been shown to disinhibit the posterior PVT projecting neurons via a D2 receptor mechanism (Beas et al., 2018). In addition, as mentioned above the CEA GABAergic pathways to the PVT could be important in cessation of feeding under threat.

### Food Seeking Behaviors: PVT Circuitries for Homeostatic and Cognitive Signals

In contrast to consumption, which has been typically elicited by inhibition of the PVT, excitation or inhibition of the PVT neurons and pathways have been shown to mediate food seeking [also see Cheng et al. (2018) for both food seeking and consumption after activation]. Similarly, drug reward seeking can be elicited by both activation and inhibition of the PVT and its pathways (Millan et al., 2017).

Activation of glucose responsive PVT neurons stimulated sucrose-seeking (Labouèbe et al., 2016) and Fos induction patterns indicate activation of PVT neurons during food anticipatory locomotion when hungry (Nakahara et al., 2004; Angeles-Castellanos et al., 2007), palatable food anticipation when sated (Mendoza et al., 2005), and renewal of cue-induced food seeking after extinction (Anderson and Petrovich, 2017, 2018). On the other hand, medial prefrontal cortex (mPFC) inputs to the PVT and PVT-nucleus accumbens (ACB) pathways are inhibited during conditioned sucrose seeking (Otis et al., 2017, 2019), and photoinhibition of the anterior PVT and its pathway to the ACB enhanced sucrose seeking (Do-Monte et al., 2017). Interestingly, that manipulation selectivity drove responding when sucrose reward was unexpectedly omitted, in accordance with a role of the PVT in balancing food seeking versus consumption based on food availability. In that regard, PVT lesions enhanced food cue driven sign-tracking (lever directed) over goal-tracking (food cup directed) behaviors (Haight et al., 2015), and blockade of ORX-R2 in the PVT of sign-tracking prone rats reduced their sign-directed behavior (Haight et al., 2020). In contrast, the blockade of ORX-R1 in the PVT of sign-tracking prone rats decreased sign- and increased goal-directed behaviors 24 h later (Haight et al., 2020), indicating potential memory consolidation effects, similar to prior findings after systemic ORX-R1 blockade (Keefer et al., 2016). Furthermore, anterior PVT neurons were recruited when ORX-R1 were blocked systemically, which inhibited cue-induced consumption (Cole et al., 2015).

In addition to cortical inputs, hypothalamic inputs to the PVT from the arcuate nucleus AgRP (NPY/GABA) neurons have been shown to drive food seeking behaviors (Livneh et al., 2017; Wang et al., 2021). The AgRP neurons are sensitive to energy balance signals and powerfully drive food consumption, via GABA or NPY release (Krashes et al., 2013). Notably, the AgRP-PVT pathway was shown to be important for food seeking and learning about food location but not for consumption (Wang et al., 2021). Interestingly, that pathway engaged AgRP and NPY, rather than GABA signaling, consistent with the notion that different substrates underlie food seeking and consumption.

In contrast to adaptive behaviors, activation of AgRP neurons in the absence of food induced stereotypic, repetitive, seemingly compulsive behaviors (increased grooming, marble burying) in mice (Dietrich et al., 2015). The data suggested that different AgRP circuitries drive these behaviors from those driving consumption but whether they involve inputs to the PVT is not known. Nevertheless, the effect was mediated by NPY Y5 receptors, which are present in the PVT area of the thalamus (Wolak et al., 2003). Systemic NPY administration is well known to strongly enhance food motivation and wheel running in an animal model of anorexia (Flood and Morley, 1991; Nergardh et al., 2007). Thus, it is plausible that the NPY–PVT pathway may drive excessive behaviors when food is absent during extreme states of hunger, perhaps similar to behaviors observed in anorexia nervosa.

How these different excitatory and inhibitory inputs are integrated within the PVT to control food seeking and whether they mediate the effects via the same output is unknown. Accumulating evidence indicates that the PVT outputs via the ACB mediate food seeking behaviors (Choi et al., 2012; Do-Monte et al., 2017; Ong et al., 2017; Cheng et al., 2018; Campus et al., 2019; Otis et al., 2019). The PVT is a complex structure and the connections of the rostral and caudal parts are distinct (Li and Kirouac, 2012). Recently, two distinct neuronal types within the PVT have been identified based on the presence of the dopamine D2 receptors (type 1) or Galanin (type 2) and other characteristics (Gao et al., 2020), These neurons are differently distributed across rostro-caudal extent of the PVT—type 1 neurons are more abundant caudally and type 2 rostrally but they have similar distribution in the middle of the PVT —and appear to function via parallel connections with the mPFC. Importantly, the type 2 neurons control arousal via the infralimbic cortex (Gao et al., 2020).

The anterior PVT has extensive connections with the prelimbic cortex and the ventral subiculum (Vertes, 2004; Li and Kirouac, 2012), while the posterior part of the PVT is more heavily interconnected with CEA and viscerosensory areas and has been implicated in stress (Hsu et al., 2014). The posterior PVT receives inputs from the ventrolateral medulla that drive hypoglycemia (glucoprivation)-induced food seeking via the ACB core (Beas et al., 2020). Interestingly, both anterior and poster PVT are needed to resolve motivational conflict when a cue signals reward and punishment (Choi et al., 2019). How reward and stress are integrated across the anterio-posterior PVT, and how they engage the type 1 and type 2 neurons, are important questions for future research.

## Hunger, Arousal, and Stress Integration Within the PVT: It Is All About Energy

Energy metabolism is tightly coupled with feeding behavior, however, energy is required for all behaviors and cell functioning but how that information is integrated across the neural systems underlying non-feeding behaviors is not clear. Similar to hunger, stress engages arousal and energy resources. The PVT is uniquely positioned to integrate hunger, arousal, and stress and adaptively regulate behavioral choice.

In addition to guiding food seeking and consumption, the PVT is important in stress and arousal (Hsu et al., 2014; Colavito et al., 2015; Millan et al., 2017). Notably, the PVT is one of the densest outputs of the lateral hypothalamic neurons that express the neuropeptide orexin (ORX; also known as hypocretin), which is critical for wakefulness, arousal and motivation (Peyron et al., 1998; Petrovich, 2019). Furthermore, the PVT is under the strong influence of interoceptive signals. In addition to the internal signals related to energy balance, the PVT receives prominent viscerosensory information, including pain, in addition to taste (Thompson and Swanson, 2003; Kirouac, 2015; Millan et al., 2017). In turn, the PVT can communicate with cortical and striatal areas within an interconnected network to determine and produce the most adaptive behavioral output (Kirouac, 2015; Vertes et al., 2015). Through these connections, the PVT could impact behavioral, hedonic, and decision-making circuitries, as well as memory formation and recall (Petrovich, 2018a).

Stress and arousal can have a major impact on feeding behavior and energy metabolism (Hsu et al., 2014; Colavito et al., 2015). Stress can inhibit or induce eating, depending on the type of stressor and timing. Anticipatory stress inhibits eating (Petrovich et al., 2009; Petrovich, 2018a), while palatable food consumption is enhanced post stress (Adam and Epel, 2007). Arousal is essential in behavioral control, from regulation of sleep/wakefulness, to reward motivated behaviors and production of excitement or induction of aversive fear and stress states. The PVT is crucial for wakefulness (Shao et al., 2019), and that circuitry was recently shown to include inputs from ORX neurons and outputs to the ACB (Ren et al., 2018).

Feeding associated anticipatory locomotion and hunger-associated arousal require the PVT (Nakahara et al., 2004; Hua et al., 2019). ORX neurons enhance arousal in response to fasting (Yamanaka et al., 2003) and support hedonic feeding and drug reward motivation via the PVT (Choi et al., 2010, 2012; Matzeu et al., 2014). Wakefulness due to hunger requires calretinin PVT neurons that project to the bed nuclei of the stria terminalis, and that pathway was proposed to mediate stress aspects of arousal during starvation (Hua et al., 2019).

### Behavioral Prioritization Based on Internal State: Adding Feelings and Action to Taste

The PVT receives very prominent visceral sensory information, including nociception. That information can arrive directly from the brainstem (nucleus of the solitary tract, parabrachial nucleus) as well as from multiple interconnected areas—including the hypothalamus, amygdala, bed nuclei of the stria terminals, and insular cortex (Thompson and Swanson, 2003; Kirouac, 2015; Petrovich, 2018a). An important function of the PVT is to appropriately match the internal state of the body with behavioral and cognitive states, and dynamically adjust them to ensure animal’s survival.

Because energy is essential for survival, hunger is an aversive state. Arousal is important for the intensity of affective states that accompany motivated behaviors. The visceral sensory (interoceptive) information has been historically associated with affect and emotion. More recently, the visceral processing neural network has been implicated in biasing many higher-order functions in humans, including the concept of self (Critchley and Harrison, 2013). Thus, in addition to guiding behavior, the PVT may be important for translating the meaning of internal states in terms of animals’ perception, cognition, and affect, as well as an overall sense of well being.

## Comparison to Previously Proposed PVT Functions

The PVT role in the adaptive control of feeding behavior that was put forward in this perspective relates to previously proposed functions for this brain area. The PVT has been hypothesized to play a critical role in wake control (Shao et al., 2019), and wakefulness and arousal are essential in the control of feeding behavior. At the minimum an animal needs to be awake in order to be able to forage and consume food, and vigor can enhance these behaviors. On the other hand, vigilance and arousal due to stress or approaching danger may shut down feeding. How the PVT integrates information about wakefulness and arousal with food foraging and consumption in an adaptive manner is an exciting area of future research.

Here, it was proposed that the PVT circuitry resolves competition between feeding and other survival behaviors, and guides switching between food seeking and consumption. The functional circuitry highlighted here was built upon the anatomical connections originally outlined by Kirouac in the context of behavioral control (Kirouac, 2015). The proposed adaptive control of feeding is related to the PVT function in resolving motivational conflicts (McGinty and Otis, 2020; McNally, 2021) and in gating which reward motivated behaviors are expressed (Haight and Flagel, 2014; Millan et al., 2017). The PVT was previously identified as critical to individual differences in Pavlovian conditioned responses (sign- vs. goal-tracking) and was postulated to underlie multiple forms of stimulus-reward learning (Haight and Flagel, 2014).

Similar to food seeking and consumption, the PVT is important for drug seeking and intake of at least some drugs (Matzeu et al., 2014; Millan et al., 2017). It is also important in drug addiction and relapse, as well as aversive states associated with drug withdrawal (Millan et al., 2017). It has been conceptualized that the PVT mediates appetitive motivation in feeding and drug addiction through its extensive connectional network (Millan et al., 2017). In agreement with the idea that common PVT substrates mediate natural and drug motivation, hunger and satiety signals have been shown to impact drug seeking and self-stimulation via the PVT (James et al., 2010; Choudhary et al., 2018; Chisholm et al., 2021). Similarly, an addiction mechanism that was suggested to involve impairments in response inhibition and PVT connectivity with the ventromedial prefrontal cortex (Huang et al., 2018) may also underlie maladaptive hedonic eating.

In addition to reward, the PVT mediates behavioral control in the context of stress (Hsu et al., 2014) and has been proposed to be a key part of the emotional processing network (Barson et al., 2020). Motivated behaviors are accompanied by affect and as discussed briefly in above section “Behavioral Prioritization Based on Internal State: Adding Feelings and Action to Taste” it is adaptive for hunger and satiety to be associated with negative and positive valence states, respectively. The PVT is well positioned to interpret interoceptive signals and affect in the context of feeding and other survival drives, and to integrate that information across behavioral and cognitive networks.

## Concluding Remarks

This perspective presented a framework for the PVT function in the control of feeding behavior within the context of energy balance and survival mechanisms, and discussed recent progress in identifying distinct PVT functional circuitries (Figure 1). That progress has been significant but has also revealed many pressing questions: Which specific PVT subsystems (cells and circuits) control different components of feeding behavior (seeking vs. consumption)? Do common or different PVT substrates mediate homeostatic, hedonic, and cognitive feeding and how are those systems regulated under stress? Do PVT outputs function in parallel or do they overlapping and where? Are there sex differences in the PVT circuits and their function? The conceptual framework presented here could provide a starting point in addressing these questions.

## Data Availability Statement

The original contributions presented in the study are included in the article, further inquiries can be directed to the corresponding author/s.

## Author Contributions

The author confirms being the sole contributor of this work and has approved it for publication.

## Conflict of Interest

The author declares that the research was conducted in the absence of any commercial or financial relationships that could be construed as a potential conflict of interest.

## References

[B1] Adam T. C. Epel E. S. (2007). Stress, eating and the reward system. *Physiol. Behav.* 91 449–458. 10.1016/j.physbeh.2007.04.011 17543357

[B2] Anderson L. C. Petrovich G. D. (2017). Sex specific recruitment of a medial prefrontal cortex-hippocampal-thalamic system during context-dependent renewal of responding to food cues in rats. *Neurobiol. Learn. Mem.* 139 11–21. 10.1016/j.nlm.2016.12.004 27940080 PMC5334368

[B3] Anderson L. C. Petrovich G. D. (2018). Distinct recruitment of the hippocampal, thalamic, and amygdalar neurons projecting to the prelimbic cortex in male and female rats during context-mediated renewal of responding to food cues. *Neurobiol. Learn. Mem.* 150 25–35. 10.1016/j.nlm.2018.02.013 29496643 PMC5893354

[B4] Angeles-Castellanos M. Mendoza J. Escobar C. (2007). Restricted feeding schedules phase shift daily rhythms of c-Fos and proteín Per1 immunoreactivity incorticolimbic regions in rats. *Neuroscience* 144 344–355. 10.1016/j.neuroscience.2006.08.064 17045749

[B5] Arcelli P. Frassoni C. Regondi M. C. De Biasi S. Spreafico R. (1997). GABAergic neurons in mammalian thalamus: A marker of thalamic complexity? *Brain Res. Bull.* 42 27–37. 10.1016/S0361-9230(96)00107-4 8978932

[B6] Barson J. R. Mack N. R. Gao W.-J. (2020). The paraventricular nucleus of the thalamus is an important node in the emotional processing network. *Front. Behav. Neurosci.* 14:598469. 10.3389/fnbeh.2020.598469 33192373 PMC7658442

[B7] Beas B. S. Gu X. Leng Y. Koita O. Rodriguez-Gonzalez S. Kindel M. (2020). A ventrolateral medulla-midline thalamic circuit for hypoglycemic feeding. *Nat. Commun.* 11:6218. 10.1038/s41467-020-19980-7 33277492 PMC7719163

[B8] Beas B. S. Wright B. J. Skirzewski M. Leng Y. Hyun J. H. Koita O. (2018). The locus coeruleus drives disinhibition in the midline thalamus via a dopaminergic mechanism. *Nat. Neurosci.* 21 963–973. 10.1038/s41593-018-0167-4 29915192 PMC6035776

[B9] Bhatnagar S. Dallman M. F. (1999). The paraventricular nucleus of the thalamus alters rhythms in core temperature and energy balance in a state-dependent manner. *Brain Res.* 851 66–75. 10.1016/S0006-8993(99)02108-3 10642829

[B10] Cai H. Haubensak W. Anthony T. E. Anderson D. J. (2014). Central amygdala PKC-δ+ neurons mediate the influence of multiple anorexigenic signals. *Nat. Neurosci.* 17 1240–1248. 10.1038/nn.3767 25064852 PMC4146747

[B11] Campus P. Covelo I. R. Kim Y. Parsegian A. Kuhn B. N. Lopez S. A. (2019). The paraventricular thalamus is a critical mediator of top-down control of cue-motivated behavior in rats. *eLife* 8:e49041. 10.7554/eLife.49041.046 PMC6739869 31502538

[B12] Cenquizca L. A. Swanson L. W. (2006). Analysis of direct hippocampal cortical field CA1 axonal projections to diencephalon in the rat. *J. Comp. Neurol.* 497 101–114. 10.1002/cne.20985 16680763 PMC2570652

[B13] Cheng J. Wang J. Ma X. Ullah R. Shen Y. Zhou U.-D. (2018). Anterior paraventricular thalamus to nucleus accumbens projection is involved in feeding Behavior in a novel environment. *Front. Mol. Neurosci.* 11:202. 10.3389/fnmol.2018.00202 29930498 PMC5999750

[B14] Chisholm A. Rizzo D. Fortin E. Moman V. Quteishat N. Romano A. (2021). Assessing the role of corticothalamic and thalamo-accumbens projections in the augmentation of heroin seeking in chronically food-restricted rats. *J. Neurosci.* 41 354–365. 10.1523/JNEUROSCI.2103-20.2020 33219004 PMC7810659

[B15] Choi D. L. Davis J. F. Fitzerald M. E. Benoit S. C. (2010). The role of orexin-A in food motivation, reward-based feeding behavior and food-induced neuronal activation in rats. *Neuroscience* 167 11–20. 10.1016/j.neuroscience.2010.02.002 20149847

[B16] Choi D. L. Davis J. F. Magrisso I. J. Fitzerald M. E. Lipton J. W. Benoit S. C. (2012). Orexin signaling in the paraventricular thalamic nucleus modulates mesolimbic dopamine and hedonic feeding in the rat. *Neuroscience* 210 243–248. 10.1016/j.neuroscience.2012.02.036 22433299 PMC3791334

[B17] Choi E. A. Jean-Richard-Dit-Bressel P. Clifford C. W. G. Mcnally G. P. (2019). Paraventricular thalamus controls behavior during motivational conflict. *J. Neurosci.* 39 4945–4958. 10.1523/JNEUROSCI.2480-18.2019 30979815 PMC6670259

[B18] Choi E. A. McNally G. P. (2017). Paraventricular thalamus balances danger and reward. *J. Neurosci.* 37 3018–3029. 10.1523/JNEUROSCI.3320-16.2017 28193686 PMC6596734

[B19] Choudhary A. G. Somalwar A. R. Sagarkar S. Rale A. Sakharkar A. Subhedar N. K. (2018). CART neurons in the lateral hypothalamus communicate with the nucleus accumbens shell via glutamatergic neurons in paraventricular thalamic nucleus to modulate reward behavior. *Brain Struct. Funct.* 223 1313–1328. 10.1007/s00429-017-1544-6 29116427

[B20] Colavito V. Tesoriero C. Wirtu A. T. Grassi-Zucconi G. Bentivoglio M. (2015). Limbic thalamus and state-dependent behavior: the paraventricular nucleus of the thalamic midline as a node in circadian timing and sleep/wake-regulatory networks. *Neurosci. Biobehav. Rev.* 54 3–17. 10.1016/j.neubiorev.2014.11.021 25479103

[B21] Cole S. Mayer H. S. Petrovich G. D. (2015). Orexin/hypocretin-1 receptor antagonism selectively reduces cue-induced feeding in sated rats and recruits medial prefrontal cortex and thalamus. *Sci. Rep.* 5:16143. 10.1038/srep16143 26536818 PMC4633617

[B22] Critchley H. D. Harrison N. A. (2013). Visceral influences on brain and behavior. *Neuron* 77 624–638. 10.1016/j.neuron.2013.02.008 23439117

[B23] Culbert K. M. Sisk C. L. Klump K. L. (2021). A narrative review of sex differences in eating disorders: Is there a biological basis? *Clin. Ther.* 43 95–111. 10.1016/j.clinthera.2020.12.003 33375999 PMC7902379

[B24] Dietrich M. O. Zimmer M. R. Bober J. Horvath T. L. (2015). Hypothalamic Agrp neurons drive stereotypic behaviors beyond feeding. *Cell* 160 1222–1232. 10.1016/j.cell.2015.02.024 25748653 PMC4484787

[B25] Do-Monte F. H. Minier-Toribio A. Quiñones-Laracuente K. Medina-Colón E. M. Quirk G. J. (2017). Thalamic regulation of sucrose seeking during unexpected reward omission. *Neuron* 94 388–400. 10.1016/j.neuron.2017.03.036 28426970 PMC5484638

[B26] Do-Monte F. H. Quiñnes-Laracuente K. Quirk G. J. (2015). A temporal shift in the circuits mediating retrieval of fear memory. *Nature* 519 461–463. 10.1038/nature14030 25600268 PMC4376623

[B27] Douglass A. M. Kucukdereli H. Ponserre M. Markovic M. Gründemann J. Strobel C. (2017). Central amygdala circuits modulate food consumption through a positive-valence mechanism. *Nat. Neurosci.* 20 1384–1394. 10.1038/nn.4623 28825719

[B28] Flood J. F. Morley J. E. (1991). Increased food intake by neuropeptide Y is due to an increased motivation to eat. *Peptides* 12 1329–1332. 10.1016/0196-9781(91)90215-B 1815219

[B29] Gao C. Leng Y. Ma J. Rooke V. Rodriguez-Gonzalez S. Ramakrishnan C. (2020). Two genetically, anatomically and functionally distinct cell types segregate across anteroposterior axis of paraventricular thalamus. *Nat. Neurosci.* 23 217–228. 10.1038/s41593-019-0572-3 31932767 PMC7007348

[B30] Haight J. L. Campus P. Maria-Rios C. E. Johnson A. M. Klumpner M. S. Kuhn B. N. (2020). The lateral hypothalamus and orexinergic transmission in the paraventricular thalamus promote the attribution of incentive salience to reward-associated cues. *Psychopharmacology* 237 3741–3758. 10.1007/s00213-020-05651-4 32852601 PMC7960144

[B31] Haight J. L. Flagel S. B. (2014). A potential role for the paraventricular nucleus of the thalamus in mediating individual variation in Pavlovian conditioned responses. *Front. Behav. Neurosci.* 8:79. 10.3389/fnbeh.2014.00079 24672443 PMC3953953

[B32] Haight J. L. Fraser K. M. Akil H. Flagel S. B. (2015). Lesions of the paraventricular nucleus of the thalamus differentially affect sign- and goal-tracking conditioned responses. *Eur. J. Neurosci.* 42 2478–2488. 10.1111/ejn.13031 26228683 PMC4596770

[B33] Hardaway J. A. Halladay L. R. Mazzone C. M. Kash T. L. (2019). Central amygdala prepronociceptin-expressing neurons mediate palatable food consumption and reward. *Neuron* 102 1037–1052. 10.1016/j.neuron.2019.03.037 31029403 PMC6750705

[B34] Hsu D. T. Kirouac G. J. Zubieta J.-K. Bhatnagar S. (2014). Contributions of the paraventricular thalamic nucleus in the regulation of stress, motivation, and mood. *Front. Behav. Neurosci.* 8:73. 10.3389/fnbeh.2014.00073 24653686 PMC3949320

[B35] Hua R. Wang X. Chen X. Wang X. Huang P. Li P. (2019). Calretinin neurons in the midline thalamus modulate starvation-induced arousal. *Curr. Biol.* 28 3948–3959. 10.1016/j.cub.2018.11.020 30528578

[B36] Huang A. S. Mitchell J. A. Haber S. N. Alia-Klein N. Goldstein R. Z. (2018). The thalamus in drug addiction: from rodents to humans. *Philos. Trans. R. Soc. B* 373:20170028. 10.1098/rstb.2017.0028 29352027 PMC5790826

[B37] James M. H. Charnley J. L. Jones E. Levi E. M. Yeoh J. W. Flynn J. R. (2010). Cocaine- and amphetamine-regulated transcript (CART) signaling within the paraventricular thalamus modulates cocaine-seeking behaviour. *PLoS One* 5:e12980. 10.1371/journal.pone.0012980 20886038 PMC2944892

[B38] Keefer S. E. Cole S. Petrovich G. D. (2016). Orexin/hypocretin receptor 1 signaling mediates Pavlovian cue-food conditioning and extinction. *Physiol. Behav.* 162 27–36. 10.1016/j.physbeh.2016.02.042 26945612 PMC4899305

[B39] Kelley A. E. Baldo B. A. Pratt W. E. (2005). A proposed hypothalamic–thalamic–striatal axis for the integration of energy balance, arousal, and food reward. *J. Comp. Neurol.* 493 72–85. 10.1002/cne.20769 16255002

[B40] Kirouac G. J. (2015). Placing the paraventricular nucleus of the thalamus within the brain circuits that control behavior. *Neurosci. Biobehav. Rev.* 56 315–329. 10.1016/j.neubiorev.2015.08.005 26255593

[B41] Krashes M. J. Shah B. P. Koda S. Lowell B. B. (2013). Rapid versus delayed stimulation of feeding by the endogenously released AgRP neuron mediators GABA, NPY, and AgRP. *Cell Metab.* 18 588–595. 10.1016/j.cmet.2013.09.009 24093681 PMC3822903

[B42] Labouèbe G. Boutrel B. Tarussio D. Thorens B. (2016). Glucose-responsive neurons of the paraventricular thalamus control sucrose-seeking behavior. *Nat. Neurosci.* 19 999–1002. 10.1038/nn.4331 27322418 PMC4964931

[B43] Li S. Kirouac G. J. (2012). Sources of inputs to the anterior and posterior aspects of the paraventricular nucleus of the thalamus. *Brain Struct. Funct.* 217 257–273. 10.1007/s00429-011-0360-7 22086160

[B44] Livneh Y. Ramesh R. N. Burgess C. R. Levandowski K. M. Madara J. C. Fenselau H. (2017). Homeostatic circuits selectively gate food cue responses in insular cortex. *Nature* 546 611–616. 10.1038/nature22375 28614299 PMC5577930

[B45] Matzeu A. Zamora-Martinez E. R. Martin-Fardon R. (2014). The paraventricular nucleus of the thalamus is recruited by both natural rewards and drugs of abuse: recent evidence of a pivotal role for orexin/hypocretin signaling in this thalamic nucleus in drug-seeking behavior. *Front. Behav. Neurosci.* 8:117. 10.3389/fnbeh.2014.00117 24765071 PMC3982054

[B46] McNally G. P. (2021). Motivational competition and the paraventricular thalamus. *Neurosci. Biobehav. Rev.* 125 193–207. 10.1016/j.neubiorev.2021.02.021 33609570

[B47] Mendoza J. Angeles-Castellanos M. Escobar C. (2005). A daily palatable meal without food deprivation entrains the suprachiasmatic nucleus of rats. *Eur. J. Neurosci.* 22 2855–2862. 10.1111/j.1460-9568.2005.04461.x 16324120

[B48] McGinty J. F. Otis J. M. (2020). Heterogeneity in the paraventricular thalamus: the traffic light of motivated behaviors. *Front. Behav. Neurosci.* 14:590528. 10.3389/fnbeh.2020.590528 33177999 PMC7596164

[B49] Millan E. Z. Ong Z. Y. Mcnally G. P. (2017). Paraventricular thalamus: gateway to feeding, appetitive motivation, and drug addiction. *Prog. Brain Res.* 235 113–137. 10.1016/bs.pbr.2017.07.006 29054285

[B50] Nakahara K. Fukui K. Murakami N. (2004). Involvement of thalamic paraventricular nucleus in the anticipatory reaction under food restriction in the rat. *J. Vet. Med. Sci.* 66 1297–1300. 10.1292/jvms.66.1297 15528870

[B51] Nergardh R. Ammar A. Brodin U. Bergström J. Scheurink A. Södersten P. (2007). Neuropeptide Y facilitates activity-based-anorexia. *Psychoneuroendocrinology* 32 493–502. 10.1016/j.psyneuen.2007.03.002 17467917

[B52] Ong Z. Y. Liu J.-J. Pang Z. O. Grill H. J. (2017). Paraventricular thalamic control of food Intake and reward: role of Glucagon-Like Peptide-1 receptor signaling. *Neuropharmacology* 42 2387–2397. 10.1038/npp.2017.150 28811669 PMC5645740

[B53] Otis J. M. Namboodiri V. M. K. Matan A. M. Voets E. S. Mohorn E. P. Kosyk O. (2017). Prefrontal cortex output circuits guide reward seeking through divergent cue encoding. *Nature* 543 103–107. 10.1038/nature21376 28225752 PMC5772935

[B54] Otis J. M. Zhu M. H. Namboodiri V. M. K. Rodriguez-Romaguera J. Stuber G. D. (2019). Paraventricular thalamus projection neurons integrate cortical and hypothalamic signals for cue-reward processing. *Neuron* 103 423–431. 10.1016/j.neuron.2019.05.018 31196673 PMC6773659

[B55] Penzo M. A. Robert V. Tucciarone J. De Bundel D. Wang M. Van Aelst L. (2015). The paraventricular thalamus controls a central amygdala fear circuit. *Nature* 519 455–459. 10.1038/nature13978 25600269 PMC4376633

[B56] Petrovich G. D. (2018a). Feeding behavior survival circuit: anticipation and competition. *Curr. Opin. Behav. Sci.* 24 137–142. 10.1016/j.cobeha.2018.09.007 31086808 PMC6510508

[B57] Petrovich G. D. (2018b). Lateral hypothalamus as a motivation-cognition interface in the control of feeding behavior. *Front. Syst. Neurosci.* 12:14. 10.3389/fnsys.2018.00014 29713268 PMC5911470

[B58] Petrovich G. D. (2019). “Orexins and control of feeding by learned cues,” in *The Orexins/Hypocretins System: Functional Roles and Therapeutic Potential*, eds Fadel J. R. Burk J. A. (Cambridge, MA: Academic Press), 85–98. 10.1016/B978-0-12-813751-2.00004-8

[B59] Petrovich G. D. Ross C. A. Mody P. Holland P. C. Gallagher M. (2009). Central but not basolateral amygdala is critical for control of feeding by aversive conditioned cues. *J. Neurosci.* 29 15205–15212. 10.1523/JNEUROSCI.3656-09.2009 19955373 PMC3321540

[B60] Peyron C. Tighe D. K. Van Den Pol A. N. De Lecea L. Heller H. C. Sutcliffe J. G. (1998). Neurons containing hypocretin (orexin) project to multiple neuronal systems. *J. Neurosci.* 18 9996–10015. 10.1523/JNEUROSCI.18-23-09996.1998 9822755 PMC6793310

[B61] Quigley J. A. Logsdon M. K. Turner C. A. Gonzalez I. L. Leonardo N. B. Becker J. B. (2021). Sex differences in vulnerability to addiction. *Neuropharmacology* 187:108491. 10.1016/j.neuropharm.2021.108491 33567305 PMC7979496

[B62] Ren S. Wang Y. Yue F. Cheng X. Dang R. Qiao Q. (2018). The paraventricular thalamus is a critical thalamic area for wakefulness. *Science* 362 429–434. 10.1126/science.aat2512 30361367

[B63] Reppucci C. J. Petrovich G. D. (2018). Neural substrates of fear-induced hypophagia in male and female rats. *Brain Struct. Funct.* 223 2925–2947. 10.1007/s00429-018-1668-3 29704225

[B64] Shao Y.-F. Lin J.-S. Hou Y.-P. (2019). Paraventricular thalamus as a major thalamic structure for wake control. *Neurosci. Bull.* 35 946–948. 10.1007/s12264-019-00364-x 30879175 PMC6754474

[B65] Stratford T. R. Wirtshafter D. (2013). Injections of muscimol into the paraventricular thalamic nucleus, but not mediodorsal thalamic nuclei, induce feeding in rats. *Brain Res.* 1490 128–133. 10.1016/j.brainres.2012.10.043 23111346 PMC3529785

[B66] Swanson L. W. (2018). Brain maps 4.0—Structure of the rat brain: an open access atlas with global nervous system nomenclature ontology and flatmaps. *J. Comp. Neurol.* 526 935–943. 10.1002/cne.24381 29277900 PMC5851017

[B67] Thompson R. H. Swanson L. W. (2003). Structural characterization of a hypothalamic visceromotor pattern generator network. *Brain Res. Rev.* 41 153–202. 10.1016/s0165-0173(02)00232-1 12663080

[B68] Veldhuizen M. G. Farruggia M. C. Gao X. Nakamura Y. Green B. G. Small D. M. (2020). Identification of an amygdala–thalamic circuit that acts as a central gain mechanism in taste perceptions. *J. Neurosci.* 40 5051–5062. 10.1523/JNEUROSCI.2618-19.2020 32371606 PMC7314406

[B69] Vertes R. P. (2004). Differential projections of the infralimbic and prelimbic cortex in the rat. *Synapse* 51 32–58. 10.1002/syn.10279 14579424

[B70] Vertes R. P. Linley S. B. Hoover W. B. (2015). Limbic circuitry of the midline thalamus. *Neurosci. Biobehav. Rev.* 54 89–107. 10.1016/j.neubiorev.2015.01.014 25616182 PMC4976455

[B71] Wang C. Zhou W. He Y. Yang T. Xu P. Yang Y. (2021). AgRP neurons trigger long-term potentiation and facilitate food seeking. *Transl. Psychiatry* 11:11. 10.1038/s41398-020-01161-1 33414382 PMC7791100

[B72] Wolak M. L. Dejoseph M. R. Cator A. D. Mokashi A. S. Brownfield M. S. Urban J. H. (2003). Comparative distribution of neuropeptide Y Y1 and Y5 receptors in the rat brain by using immunohistochemistry. *J. Comp. Neurol.* 464 285–311. 10.1002/cne.10823 12900925

[B73] Yamanaka A. Beuckmann C. T. Willie J. T. Hara J. Tsujino N. Mieda M. (2003). Hypothalamic orexin neurons regulate arousal according to energy balance in mice. *Neuron* 38 701–713. 10.1016/S0896-6273(03)00331-3 12797956

[B74] Zhang J. Chen D. Sweeney P. Yang Y. (2020). An excitatory ventromedial hypothalamus to paraventricular thalamus circuit that suppresses food intake. *Nat. Commun.* 11:6326. 10.1038/s41467-020-20093-4 33303759 PMC7728757

[B75] Zhang X. van den Pol A. N. (2017). Rapid binge-like eating and body weight gain driven by zona incerta GABA neuron activation. *Science* 356 853–859. 10.1126/science.aam7100 28546212 PMC6602535

